# “Like one part of a puzzle” — individualized aromatherapy for women with gynecological cancers in aftercare: results from a qualitative-focused mixed-methods study

**DOI:** 10.1007/s00520-022-07543-z

**Published:** 2022-12-23

**Authors:** Judith Czakert, Wiebke Stritter, Sarah B. Blakeslee, Jacek P. Grabowski, Jalid Sehouli, Georg Seifert

**Affiliations:** 1 Universitätsmedizin Berlin, corporate member of Freie Universität Berlin and Humboldt-Universität zu Berlin, Department of Pediatric Oncology and Hematology, Integrative Medicine in Pediatric Oncology, Berlin, Germany; 2 Universitätsmedizin Berlin, corporate member of Freie Universität Berlin and Humboldt-Universität zu Berlin, Department of Gynecology, Berlin, Germany

**Keywords:** Integrative oncology, Symptom control, Gynecological cancer, Breast cancer, Cancer survivors, Long-term care, Aftercare, Aromatherapy, Essential oil, Cancer burden, Qualitative research

## Abstract

**Background:**

Gynecological cancer(s), including breast cancer patients in aftercare and survivors, need supportive strategies to cope with symptoms that are adapted to their individual needs and circumstances. Aromatherapy has potential to be such strategy, but (qualitative) empirical research taking users’ own views into consideration about the potential and challenge of aromatherapy is lacking.

**Purpose:**

The purpose of the study is to gain insights from individualized aromatherapy as a supportive care treatment, regarding their use and evaluation by women with gynecological cancers in aftercare.

**Methods:**

We conducted a study with a mixed-methods design, focused on qualitative research. Five essential oil products were given to 18 participants to apply individually over a 4-week period. After the intervention, qualitative semi-structured interviews were conducted. Further, we documented and assessed symptomatic burdens of the women (MYMOP2) before and after intervention quantitatively.

**Results:**

Aromatherapy was customized by the participants according to their needs. It showed potential for relief of symptomatic burdens — especially nausea, peripheral neuropathy, pain, and sleep. Additionally, opportunities emerged to indirectly affect symptomatic burdens. These developed out of new coping strategies (e.g., sleep routines) or by combining with existing strategies (e.g., meditation). Furthermore, aromatherapy was successfully used to promote well-being and encourage mindfulness.

**Conclusion:**

Our findings demonstrated the potential of aromatherapy as a supportive treatment modality that can be used as a kind of toolbox. Challenges, such as individual odor aversions and intolerances, and limitations due to medication or illness should be considered in future aromatherapy research.

**Supplementary information:**

The online version contains supplementary material available at 10.1007/s00520-022-07543-z.

## Background

Women with gynecological cancers, including breast cancer, must often contend with cancer- and therapy-related side effects such as pain, fatigue, stress, sleep disturbances, cognitive impairment, neuropathy, psychological distress, changes in sexuality [1–3], or clusters of several symptoms [3]. Symptomatic side effects usually do not vanish directly post chemotherapy but continue into aftercare — often into the survivor phase [1, 2, 4]. Side effects of cancer can have strong negative impacts on women’s daily lives and general well-being [3, 5]. Tools and care given by health care professionals for women’s persisting symptomatic burdens have high relevance for those affected — for instance, as a part of individualized survivorship care plans (SCPs) [4]. However, enduring symptoms after cancer treatment often remain undertreated [2, 6]. The desire for symptom relief in daily life might explain the draw of complementary and integrative medicine (CIM)^1^ approaches for women with gynecological cancers and breast cancer [1, 7]. The relevance of our research results from these points and can be summarized as follows: The increasing trend toward CIM in women’s cancer care coupled with the need for evidence-based, individually adaptable strategies to cope with such symptomatic burdens necessitates empirical research of aromatherapy as one CIM method. Aromatherapy has gained growing public interest as a supportive therapeutic treatment for various health-related reasons and as a wellness application, as is evident from its media presence. Accordingly, interest in aromatherapy as a supportive therapy for symptomatic burdens due to cancer (treatment) is increasing. Empirical research results reinforce the potential to manage symptoms and improve general well-being of cancer patients by aromatherapy [8, 9]. However, there is still a lack of target group-specific research to consider user perspectives and experiences.

Additionally, clarity about what aromatherapy actually means is needed [10].

### Understanding of aromatherapy

Aromatherapy is not always well-defined in health-related research [10]. For this work, we use the definition of the National Center for Complementary and Integrative Health (NIH) which explains aromatherapy as “the use of essential oils from plants (flowers, herbs, or trees) as a complementary health approach” (https://www.nccih.nih.gov/health/aromatherapy. 04.04.2022).

Essential oils are multi-substance mixtures of plant components, such as bark, resin, flowers, leaves, and fruit peels. They are obtained through various pressing and distillation processes [11]. Each essential oil is distinct, as the chemical compositions depend on the conditions under which the corresponding plants were grown [12]. This presents a challenge to aromatherapy research, as exact replications of interventions are quite impossible [10].

### What is known about aromatherapy

Although the complex interaction of how aromatherapy works is not conclusively clarified, the following assumptions form the basis of this work:

Essential oils can be divided into three types of absorption: via the skin and mucous membranes, olfactory and oral [13]. In medical and nursing contexts, absorption mostly works through the olfactory via inhalation or room scenting, or through skin and mucous membranes via percutaneous massages and embrocation [14].

A distinction can be made between psychological and pharmacological mechanisms of aromatherapy. The psychological effect — triggered by the olfactory intake of the essential oils — can be divided into three areas that control the individual scent reaction: (1) subjective evaluation (hedonic valence); (2) conditioning (semantic mechanism); and [3] expectancy (placebo effect) [15, 16]. Hence, the personality and the cultural imprint of the person smelling play a decisive role on the specific, olfactory-triggered effect. The pharmacological effect, however, is based on the absorption of the essential oil components via the skin and mucous membranes, and the specific composition of the respective oil [15] and acts similar in each user. Depending on the mode of application (skin vs. olfactory absorption), essential oils can trigger significantly different effects [17]. Under natural conditions, separate mechanisms of action can hardly be differentiated [15] and the individual responses to smell are difficult to predict. Nevertheless, they should be taken into account in research designs [10].

### Aromatherapy research in oncology

Multiple reviews explicitly refer to aromatherapy in the oncology setting: some focus on specific symptomatic complaints, such as nausea and vomiting [18], and pain [19]. Others focus on broad use of aromatherapy for symptom control [9], as a supportive treatment in cancer [8, 20], or combined with massages [21–23]. Primary studies on aromatherapy in oncology show significant improvements in sleep [24, 25], pain [26], anxiety [27], fatigue [28], and nausea and vomiting [29, 30]. In the few qualitative surveys on applications with essential oils in the context of oncological diseases, these are perceived as positive overall [31–34]. Aside from these promising findings, we previously identified a research gap [10]: women with gynecological cancers are underrepresented as a target group for aromatherapy research, as is the cancer aftercare and survivorship phase. Individually applied aromatherapy has yet to be scientifically and exploratively investigated from the patients’ point of view.

Research that explores how aromatherapy is used and understood by women post gynecological cancers and breast cancer treatment is needed. This necessitates taking special consideration of the opportunities, challenges, experiences, and attitudes that come along from the users’ subjective point of view. Due to this research gap, this study focuses on the specific target group of women with gynecological cancers and their personal experiences with aromatherapy in everyday use. Accordingly, the research aims and questions are as follows:

### Aims and research question

We intend to explore potential and challenges of individualized aromatherapy in the everyday life of women with gynecological cancers — including breast cancer — in aftercare, with focus on their experiences and perceptions. We aim to provide implications for future development of aromatherapy interventions for women after cancer treatment.

The underlying research question is:

What insights can be gained from individualized percutaneous and olfactory applications of essential oils by women with gynecological cancers and breast cancer in aftercare with regard to their use and evaluation?

The question was differentiated into two parts:

1. What potential and challenges can be identified from the participant’s experiences with the products?
2. Does the burden of symptomatic complaints change after the intervention?

## Methods

The research design was developed by an interdisciplinary and interprofessional team consisting of physicians, nursing, and public health researchers, and aromatherapists with many years of practice and experiences. To include a vast range of participant perspectives and points of view assessing a individually tailored intervention, we chose a mixed-methods approach [35] with a focus on qualitatively explored experiences. The aromatherapy intervention enabled the participants to tailor essential oil use to their individual needs and choose between different products and applications: Five selected essential oil products from Primavera® (Table 1) were compiled for each participant (*n* = 20). The selection of essential oils was made with the help of experienced aromatherapists. The underlying goal was to offer a large selection of essential oils. Products were chosen to elicit a wide range of possible effects (regarding distinct symptomatic burdens; see Table 1), consider the olfactory preferences of the participants through different fragrances, and offer various applications that cover the individual needs and circumstances of living of the participants. This was done in the spirit of an exploratory approach, designed to give the participants room to apply aromatherapy as fully and well-adapted to their lives as possible. In addition, each participant received a brochure with information on the products, use safety, listed the essential oils ingredients, the plants they have been extracted from, and the potential areas of application and possible uses (Table 1). Equipped with this background information, the participants could choose products, time/duration, and type of use over a four-week period by independently adapting them to their needs and requirements.

**Table 1:** Essential oil products for aromatherapy intervention

*PRODUCT*	*DECLARATION*	*ESSENTIAL OIL(S)*	*APPLICATION EXAMPLE*	*USES EXAMPLE*
PFEFFERMINZ (PEPPERMINT)	Cosmetic	*Mentha piperita*	Application via scent carrier, vaporization	Fatigue, nausea
GUTE LAUNE (GOOD MOOD)	Requirement item	*Citrus sinensis*, *Citrus limonum*, *Citrus aurantiifolia*	Application via scent carrier, vaporization	Fatigue, stress, nausea
ENTSPANNUNGSÖL (RELAXING OIL)	Cosmetic	*Lavandula angustifolia*, *Styrax tonkinensis*, *Cymbopogon martinii*	Body massage	Anxiety, sleep disturbances, stress
ENERGIEKICK (ENERGY KICK)	Cosmetic	*Pseudotsuga menziesii*, *Abies alba*, *Citrus paradisi*	Apply on neck, temple, wrist	Fatigue
KISSENSPRAY (PILLOW SPRAY)	Requirement item	*Lavandula angustifolia*, *Citrus aurantium*, *Vanilla planifolia*	Room/pillow spray	Anxiety, sleep disturbances, stress

### Ethical considerations

An ethical approval from the Ethical Committee of Charité – Universitätsmedizin Berlin was received (EA2/027/21) and written informed consent was obtained from all participants. During the intervention period, the participants could contact the research team in case of questions. Also, they were informed that they could withdraw from the study at any time without giving reasons.

### Design

Research questions were investigated using a mixed-methods design with a focus on a qualitative approach with an embedded quantitative part [35]. To answer part 1 of the research questions, a qualitative approach was chosen, to capture the potential and challenges of aromatherapy. Understanding the perceptions and experiences of the participants by exploring their subjective perspectives was imperative to this qualitative approach. The embedded quantitative part fulfils a supportive role [35] by giving an overview of the subjective change of symptom burden after the intervention — according to part 2 of the research questions. We used this first overview to compare the qualitative data with.

### Sample and recruitment

With the help of gatekeepers (JS and JG) in the Department for Women’s Medicine at Charité – Universitätsmedizin Berlin, and with a snowball sampling strategy, we compiled a purposive sampling (*n* = 20) with maximum variation [36] in terms of age, cancer diagnosis, and time since the last chemotherapy. Inclusion criteria were as follows: women with gynecological cancers or breast cancer in aftercare; last intensive polychemotherapy at least 6 weeks prior to study participation; German language skills; age at least 18 years old. Interested patients with known allergies to ingredients of the products were excluded.

### Data collection and analysis — quantitative research part

The quantitative data collection is based on the validated German version of the questionnaire *Measure Yourself Medical Outcome Profile* (MYMOP2) [37]: MYMOP2 is a generic patient-reported outcome tool to collect and assess the two most relevant, disease-/therapy-related symptomatic burdens of each woman before and after the intervention, respectively related to the last week. In addition, socioeconomic data and diagnostic data were collected, descriptively presented, and included in the qualitative data collection and discussion.

### Data collection and analysis — qualitative research part

After the intervention, the participants were asked about their personal experiences with the intervention via semi-structured interviews, with the aim to capture a wide spectrum of themes, perspectives, and perceptions, related to the intervention [38]. The interview guideline was developed in a team with experienced qualitative researchers based on the principles by Helfferich [39], in order to be “as open as possible, as structured as necessary” (ibid.). The guide was structured in three steps: (1) introductory topic-related question to initiate a free narrative; (2) probing inquiries; (3) and open, pre-formulated questions to address remaining topics of interest. The initial open narratives allowed the women to choose their own thematic relevance and how they speak about aromatherapy. The interview guideline was structured by the topics symptom reference, everyday application, experiences with aromatherapy, and evaluation of aromatherapy. It was developed and discussed within the whole research team and adapted after a pilot interview has been conducted.

After transcription of the interviews according to Dresing and Pehl [40], a deductive-inductive qualitative content analysis [41] was conducted using the software MAXQDA. All interviews were first summarized regarding thematic focal points in order to provide an overview and become familiar with the data. Then, interview content was sorted into a deductive category system derived from the research questions. New codes were inductively created from the content and integrated into the code system. The code system was regularly discussed and consulted with other qualitative experts (WS, SB), in order to strengthen inter-subject comprehensibility as one quality criteria of qualitative research [42].

The integration of the qualitative and quantitative research part was merged at two points: (1) content of the survey MYMOP2 was taken up during the interviews (symptomatic burdens), and (2) results were related to each other in the discussion.

## Results^2^

Twenty participants were initially included. Two dropped out after 2 weeks due to (1) private reasons and (2) no reason cited. Eighteen participants completed the study 4 to 6 weeks after the intervention with the follow-up survey of MYMOP2 and the qualitative interview. The participants’ age distribution ranged between 39 and 77; the majority were between 50 and 60 years old. The participants had breast (*n* = 6), ovarian (*n* = 11), and uterine (*n* = 1) cancers. Their last chemotherapy was between 6 months and 7 years ago. For most of the women (*n* = 6), the last chemotherapy was completed in the previous 1–2 years. One woman started with a grade IV chemotherapy after 10 days of aromatherapy. An overview about the characteristics of the participants is shown in Table 2.

**Table 2:** Characteristics of the participants

Participant	Age	Diagnosis (ovarian cancer (O), uterus cancer (U), breast cancer (B))	Time since diagnosis (< 1, 1–2, 3, 4, 5 + years)	Therapy				Symptomatic burdens (MYMOP2)
				Chemotherapy	Surgery	Radiation therapy	Maintenance therapy	
TN_01	70–80	O	1–2	X	X			(1) Peripheral neuropathy (2) Fatigue
TN_02	50–60	O	1–2	X	X		PARPi	(1) Peripheral neuropathy (2) Intestinal complaints
TN_04	50–60	O	1–2	X	X		PARPi	(1) Fatigue (2) Peripheral neuropathy
TN_05	60–70	U	4	X	X	X		(1) Pain (2) Intestinal complaints
TN_06	70–80	O	5 +	X	X			(1) Peripheral neuropathy (2) Intensified odor sensation
TN_07	60–70	O	1–2		X	X		(1) Pain (2) Peripheral neuropathy
TN_08	60–70	B	< 1	X	X	X		(1) Peripheral neuropathy (2) Fatigue
TN_09	40–50	B	4	X	X			(1) Fatigue (2) Anxiety
TN_10	50–60	O	< 1	X	X		VEGFi	(1) Rheumatism (2) Concentration problems
TN_11	50–60	B	4	X	X	X		(1) Sleeping disorders (2) Temper
TN_12	60–70	O	3	X	X			(1) Fatigue (2) Intestinal complaints
TN_13	50–60	O	5 +	X	X		PARPi	(1) Depression (2) Peripheral neuropathy
TN_14	60–70	O	3	X	X			(1) Sleeping disorders (2) Fatigue
TN_15	40–50	O	3	X	X			(1) Peripheral neuropathy (2) Depression
TN_16	50–60	O	3	X	X			(1) Peripheral neuropathy (2) Depression
TN_18	18–40	B	5 +	X	X		X	(1) Fatigue (2) Dry mucous membranes
TN _19	50–60	B	1–2		X		X	(1) Hot flushes (2) Sleeping disorders
TN_20	50–60	B	1–2		X		X	(1) Pain (2) Sleeping disorders

### Results of the MYMOP2 questionnaire

**Fig. 1:**
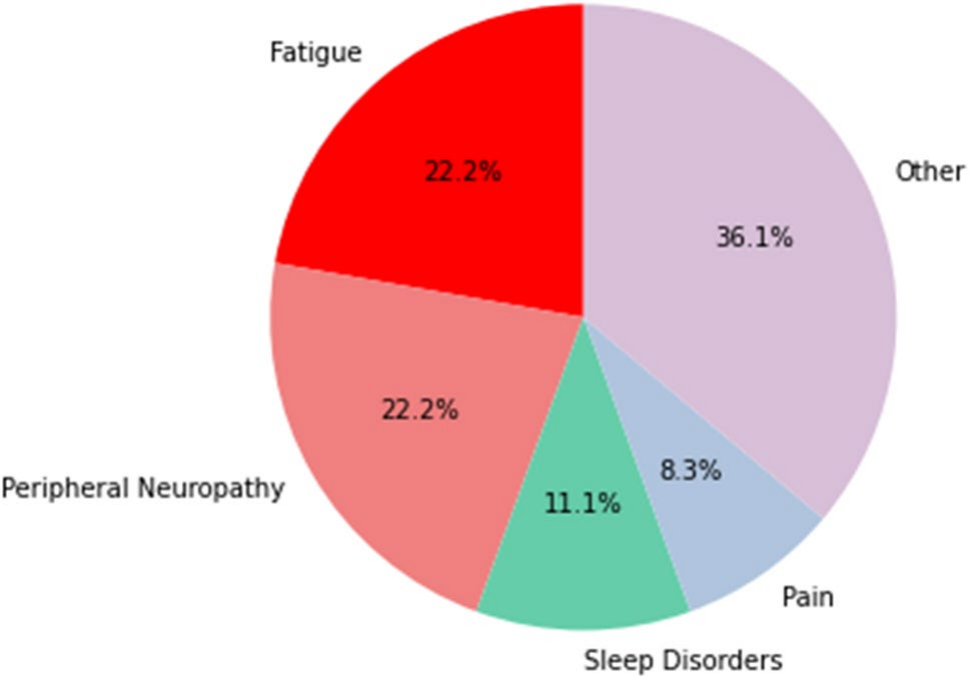
Symptom burden

**Fig. 2:**
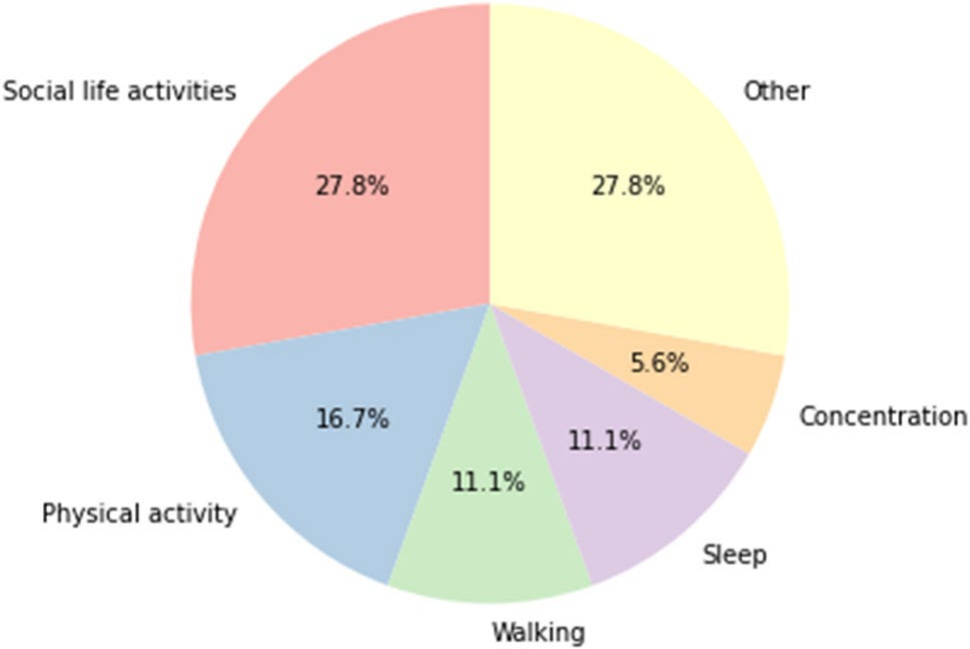
Limited activities

**Fig. 3:**
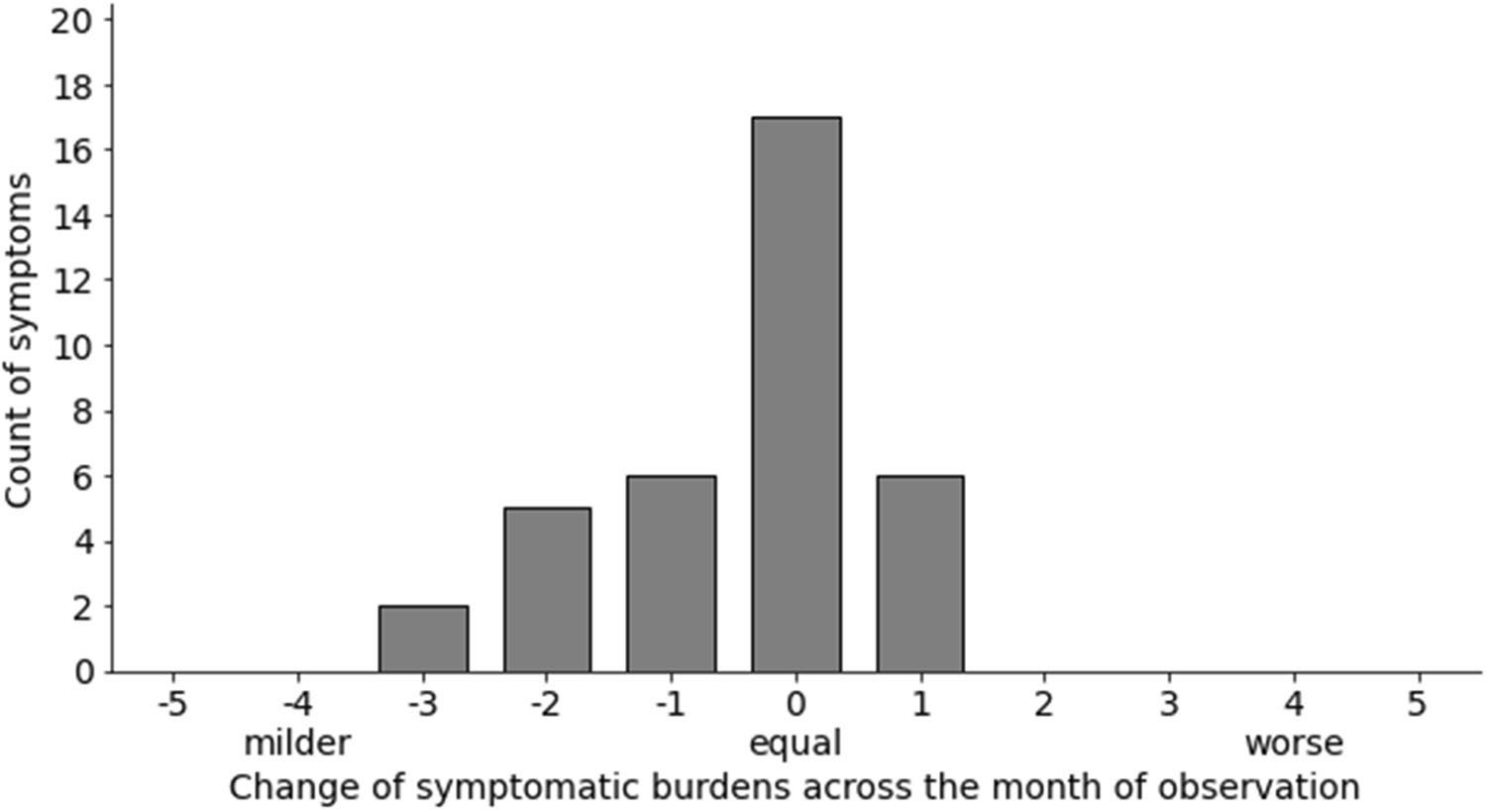
Change of symptomatic burdens

**Fig. 4:**
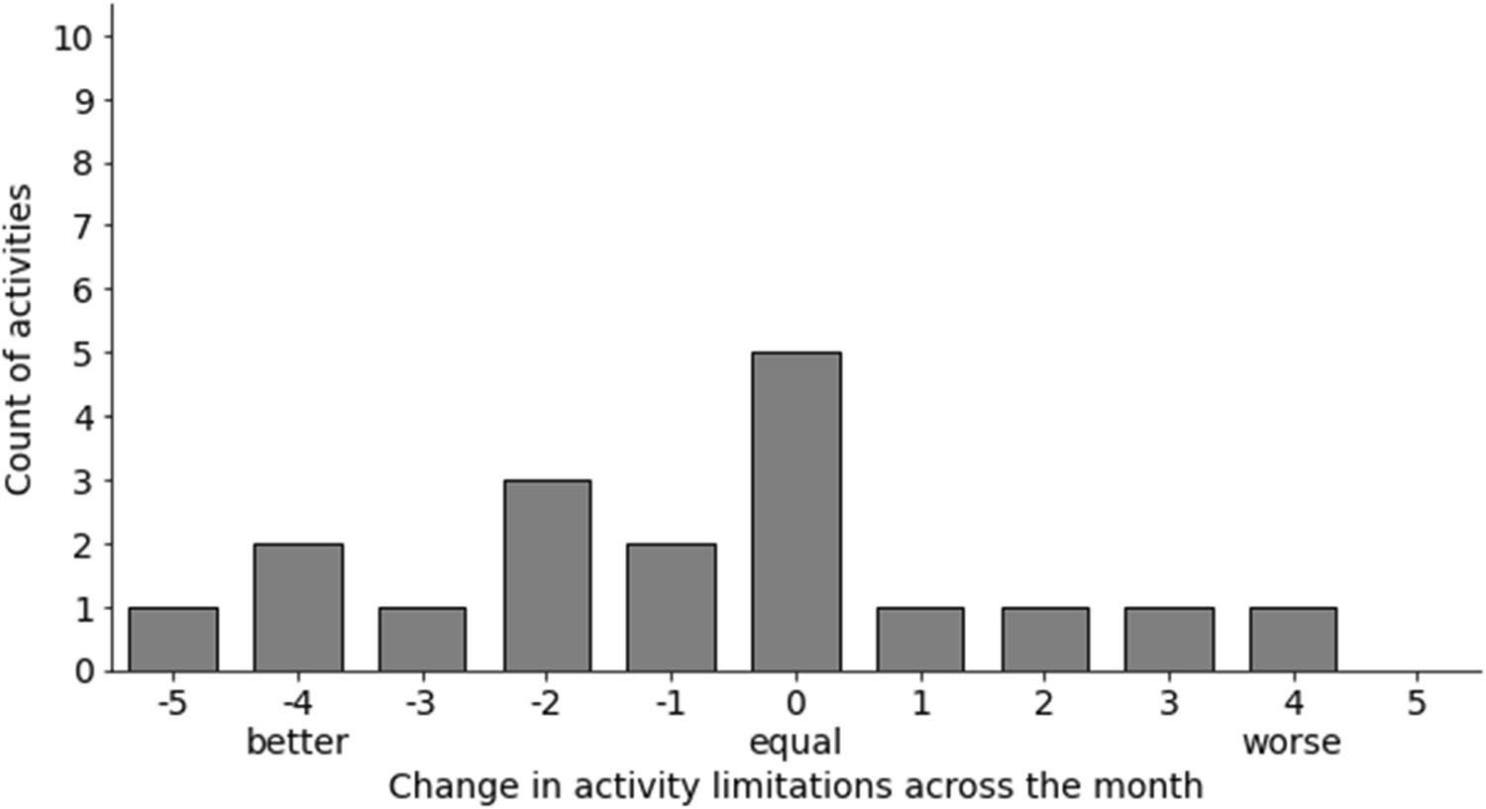
Change in activity limitations

The most relevant, therapy-related symptoms listed by the participants in the MYMOP2 questionnaire were peripheral neuropathy (*n* = 8), fatigue (*n* = 8), sleeping disorders (*n* = 4), and pain. In addition, women listed anxiety, depression, dry mucous membranes, hot flashes, intensified olfactory perception, intestinal complaints, and aggravation of rheumatism (Fig. 1). Activities that are hindered by the mentioned symptoms are shown in Fig. 2. General deterioration of social life was indicated by most women as a complication of activities resulting from the symptomatic burden. Specific individual symptoms were documented under the category “other,” and are related to nutrition, studying, or activity limitations in general. The follow-up MYMOP2 data were based on the perceptions of the participants about the same two symptoms and one limited activity they had already indicated and assessed in the baseline survey. The overall changes in symptom burdens (Fig. 3) and in limitations of activities (Fig. 4) show a tendency toward improvement. This initial outlook is augmented and contextualized by the qualitative data from the semi-structured interviews, which each lasted between 18 and 80 min.

### “It’s one part of a puzzle” — individual experience and perceptions on individualized aromatherapy

Compared to the MYMOP2 survey results, the qualitative data show a broader and deeper picture of essential oil use. Not only concerning the potential for treatment beyond improving symptom burden, general wellbeing, and activity limitations, but also the different ways that aromatherapy can be used, and the challenges, the experiences, and the accompanying attitudes. Most participants had had previous experiences with essential oils used as wellness products. Expectations of strong symptom relief through aromatherapy were rarely mentioned explicitly. However, some cases implicitly expressed disappointment signaled by a high level of expectation. Nevertheless, almost all participants were positively oriented toward aromatherapy. Individual application adapted to their needs and subjective preferences appeared to be vital for most participants’ use of the essential oil products.

The reasons for use were extremely diverse, ranging from specific and non-specific (Table 3). Characteristic features in the use of essential oils could be identified, regarding place of use, frequency of use, type of usage, and motivation, which are compiled in Table 4 (see in detail: supplement 1).

**Table 3:** Reasons to use aromatherapy

Main-codes	Sub-codes	Exemplary quotes
Non-specific reasons	**General improvement of well-being** → Doing something good (1) → “Just because I like the smell” (2) → Undirected reasons (3)	(1) *So, when you’re in the situation with cancer, then […] you naturally pull at all straws and I just try to do something good for myself. And I have to say that for me this was very, very pleasant.* (TN01) (2) *[I] use it […] because I just love the smell.* (TN02) (3) *[I] thought, just take it out right now, just do it and also do it differently, I would say. I can’t now say how, when, but I used it very often.* (TN01)
Specific reasons “Means to an end”	**Relief of symptom burden** → Symptoms reported in MYMOP (sleep (1), fatigue, peripheral neuropathy, anxiety/depression) → Other symptoms (nausea, pain (2), skin and scar care) **Coping strategies for everyday challenges** → Activation (e.g., substitute for coffee (3), rise energy level) → Stabilization → Relaxation → Distraction → Refreshment → Concentration → Mark and support transitions → Support (4)	(1) *The pillow spray. I actually used that often, because I noticed that it helped me to fall asleep*. (TN20) (2) *And I used it [Peppermint, JC] partially, I think, for massaging the places that had pain; I massaged it in.* (TN20) (3) *The Energy Kick has slowly replaced coffee, at least it has the character of a ritual, I think.* (TN08) (4) *With migraines it gives a little support in the way that I could say that the migraine isn’t so sharply painful. I don’t know if it’s possible to understand what I mean.* (TN13)

**Table 4:** Characteristics of using aromatherapy

Characteristics	Characteristic expressions	Examples/exemplary quotes
Place of use	Participants used aromatherapy mostly at home and work, but also (some) mentioned using it while travelling and on holiday	For instance, in the office, hotel rooms, car, train
Frequency of use	Overall, there is a wide variation between the poles of regularity/structure and irregularity/unstructuredness. Striking is the frequent establishment of routines. In some cases, these routines led to positive conditioning	Exemplary quote for positive conditioning: “Ah yes, feel supported. I feel fortified with the thought alone that I’m taking part in this study. Yes, […] the thought alone was helpful.” (TN09)
Type of usage	(1) Percutaneously (2) Olfactorily	(1) Often combined with massage (2) Scenting in the air and scenting via a carrier object (e.g., pillows, curtains, handkerchiefs, masks, odor sticks, candles
Motivation	(1) Commitment and legitimacy (2) Impetus from the study-framework	(1) The study-assignment legitimized participants to act in their own self-interest, and to take time for self-care: “But the stimulus of this study […] clearly released the feeling in me, that I have all the time in the world to intensively pursue and use these alternatives.” (TN12) (2) Participants described their motivation to experiment with the essential oils with words like *euphoric*, *thrilled*, *enthusiastic*, *curious*

From our analysis, we found potential, challenges, and a co-existence of both in dealing with aromatherapy (Table 5). The identified potential of aromatherapy can be summed up in its use as a tool for promoting self-care in different areas and with different approaches: for symptom control, promoting well-being, encouraging mindfulness, and developing strategies. One issue in particular to be highlighted is the interaction between potential and challenge: the topic “prior information” can have both positive and negative consequences on the perception of aromatherapy and its effects. The challenges with aromatherapy that were reported, were associated with adverse events and individual problems in regard to the essential oil products (see in detail: supplement 1).

**Table 5:** Potential and challenges of aromatherapy

Description	Characteristic expressions	Examples/descriptions
Potential		
Aromatherapy as a tool for symptom management	**Reported symptom improvement** (1) Symptoms, mentioned in MYMOP2 (2) Symptoms, not mentioned in MYMOP2 Improvement described as moderate improvement and often closely linked with increased mindfulness and a intensified focus on self-care	(1) Sleeping disorders, fatigue, pain, various manifestations of peripheral neuropathy, nausea (“The peppermint oil I use three times a day, always when I notice that I feel nauseous and then I feel free of nausea. So even now it still always works.” (TN02)) (2) Headaches, muscle pain, skin and scar care; often in combination with conventional treatment like pain-medication or other strategies, such as cold compresses, resting in the dark, or massaging the temples: “It really calmed me a bit, when I could say: Okay, relax, relax. So, I would need a little less of the medication […]. So, I found that very pleasant” (TN13)
Aromatherapy as a tool to promote well-being	(1) **Influence energy level** a. Relaxation and calming down (mostly used for this purpose: *Pillow Spray* and *Relaxing Oil*) b. Activation, invigoration, and refreshing (mostly used for this purpose: *Energy Kick, Good Mood, Peppermint*) (2) **Mood-improvement** Participants reported a feeling of security, increased optimism and good mood, feelings of happiness, and general mood-enhancing effects through aromatherapy	(1) a.) Also, but rarely, other products were used to calm down and relax, when participants recognized that a specific odor, triggered calming memories in them: “Peppermint is a smell that for me is very positively-associated with my grandmother. And even now I use it […] often and constantly, because it calms me. As I said, because there’s so much past there.” (TN13). Some participants even reported how the regular exposure to the essential oils, regardless of the products, led to a feeling of relaxation and stress relief (2) “And you feel, that you [do] something good for yourself. Yes.” (TN09)
Aromatherapy as a tool to encourage mindfulness	**Using the anchoring function of odor to be aware of the moment** → To change unwanted situations → To strengthen desired emotional states → To create personal space → To feel protected and secure → To shift focus → To foster motivation	“It’s like an anchor” (TN 09)
Aromatherapy as a tool to develop strategies	(1) Development of new strategies (2) Support of existing strategies	(1) e.g., routines, based on essential oil applications, (2) e.g., essential oil applications in conjunction with other coping strategies (such as meditation, medication, mindfulness training) or as an assistive device to soften (illness related) challenges in everyday life “There the [radiation, JC] is working internally, and then to do something about it that can work against that, like these beautiful scents, it does have a [soothing] effect.” (TN08)
Potential & Challenge		
Ambiguity of prior information	Expectations, created by product name or information may guide the perceived effects in specific directions (1) Potential: Reinforcement of desired effect(s) (2) Challenge: Perception of unexpected effects is hindered	(1) “So, I also liked this fragrance, I liked the smell of it, and I found it pleasant and then it said on it ‘Energy’ and I thought—I need energy now, so I will take energy. And then I felt energy.” (TN04)
Challenges		
Associated adverse event	(1) Association of lavender-based products with breast swelling (2) Irritation of the nasal mucosa	(1) The most serious effect reported was the association of the lavender-based products (*Pillow*, *Spray*, and *Relaxing Oil*) with swelling of both breasts after mastectomy in one participant. As a result, the lavender-based products were reduced by the participant until the swelling decreased (2) Another patient with therapy-related sensitive mucosa nose-skin stopped using the essential oil products after three weeks — except *Relaxing Oil* — due to irritated nasal mucosa
Individual Challenges	→ Difficulties in opening the bottles → Lack of time to use the products regularly → Olfactory aversion by members of the household → Olfactory aversion by the participants → Disappointment due to unfulfilled expectations	

### Interim conclusion

Despite the challenges presented, and although not all participants were able to report improvement in their specific symptoms, they all had a positive overall summary of their experiences with the essential oils — each with its own unique explanation. On this basis, it may be concluded that aromatherapy within the targeted group has more holistic potential than mere symptom management. One participant provides context to the way that aromatherapy can be used as one approach alongside a multifactorial strategy to address the symptomatic burden: “And I am still convinced that this is one part of a puzzle …” (TN12)*.*

## Discussion

Women with cancer in aftercare and as cancer survivors are particularly motivated to be proactive in their recovery and healing process (1). By offering appropriate tools suitable for everyday life, they may be better supported. In our study, we were able to identify the potential (AT as a tool for symptom management, AT as a tool to promote well-being, AT as a tool to encourage mindfulness, AT as a tool to develop strategies) and challenges (association of lavender-based products with breast swelling, irritation of the nasal mucosa, and individual challenges concerning conditions and circumstances of the users) of aromatherapy when applied as such a tool (Table 5). This takes the target groups’ personal life circumstances, needs, and preferences into consideration. The identified potential of aromatherapy requires a greater scrutiny.

### Aromatherapy: a toolbox for symptom management

The qualitative results allow insights into the potential of aromatherapy regarding symptom management. They showed mostly moderate improvements of symptom burdens such as sleeping disorders, fatigue, moderate pain, nausea, and peripheral neuropathy. This corresponds to our quantitative findings about the change of symptom burden after aromatherapy (Fig. 3) and confirms the existing evidence on general symptom improvement with aromatherapy in cancer care [8, 9, 20].

The results can be distinguished between direct and indirect impacts on symptomatic burdens:

#### Direct impacts

Due to the participant’s individual approach and use of essential oil products, their perception of direct effects can only be partially attributed to specific products and their applications. Direct effects of aromatherapy were found with (1) lavender-based products (*Pillow Spray* and *Relaxing Oil*) for improving sleep disorders; (2) *Relaxing Oil* in combination with massages against painful cramps and for activation of numb limbs in peripheral neuropathy; (3) *Peppermint* against nausea, in line with recent research about use of peppermint essential oil in oncology settings [18, 30]. Regarding the effectiveness of lavender for therapeutic purposes, research is inconclusive [43]. However, recent reviews summarize promising evidence for lavender use against sleeping disorders [44, 45]. Empirical research on the efficacy of lavender against neuropathic pain is limited; however, basic research on mice [46] and in patients with diabetes [47] has been demonstrated. More empirical research is needed to further explore the potential of lavender (and massage) for peripheral neuropathy.

#### Indirect impacts

Moreover, our findings show the great potential of aromatherapy to help cope with symptomatic burdens by promoting well-being, encouraging mindfulness, and developing individual strategies. This is in line with research about use of aromatherapy as an adjuvant treatment [20]. Individually tailored aromatherapy, autonomously adapted to needs and circumstances of the participants, has the potential to initiate and support a process of empowerment by offering an opportunity to act autonomously within the context of illness-related symptomatic burdens. Playing an active role and making free and creative decisions about health issues may enhance the sense of control [48]. Particularly in the follow-up period of aftercare, after a phase of greater dependency on medical professionals’ opinions and decisions, a sense of empowerment might gain importance [48]. The empowering character of aromatherapy is consistent with the call for greater involvement of cancer patients in health care issues [49].

### Indications for future concept development

Based on our findings, the following points should be considered for further development of aromatherapy interventions:

#### Professional guidance

Aromatherapy has the potential to give users something they urgently need for their well-being, coping, and healing, but they would not have practiced without the legitimization of the study assignment: taking time for oneself and self-care [32]. To legitimize and establish aromatherapy, it should be explicitly recommended by medical professionals as one tool that may support and develop coping strategies. In doing so, it is important that the patients obtain reliable information about aromatherapy and that there is no contraindication for their standard treatment — as is also required for CIM in general [7]. To increase motivation, recommendations on possible uses, included by the health care professionals, would provide helpful guidance on initial applications. At the same time, encouraging users to adapt the aromatherapy independently to their needs, requirements, and life circumstances has the potential to expand and increase autonomous use of the essential oils. This would benefit from consideration of individual olfactory preferences and dislikes.

#### Inclusion of individual odor preferences and aversions

Our results indicate that odor preferences and aversions have a clear impact on motivation and perceived effects. The smell of the products alone can cause strong reactions (physical or mental responses such as nausea, vomiting, reluctance or positive emotions, relaxation) and even behavioral changes (creating routines, establishing a frequency of use, conditioning effects). The effect of the odor alone is considered to be even more important than the effect of the chemical components [50]. That means, for example, “if an individual does not like the scent of lavender, she will not find it relaxing, regardless of how well and widely lavender aroma has been marketed as ‘relaxing’” [50]. Our findings support this view. Thus, it is important to take the subjective differences in the patients’ perception of odors into account, when offering aromatherapy.

#### Note and use the potential conditioning through aromatherapy

Aromatherapy could link negative experiences of cancer treatment with a specific odor. For instance, an odor could be associated with anxiety that is triggered by radiotherapy [51]. Like a negative Pavlovian response, the odor alone has the potential to trigger anxiety at a later stage. On the other hand, our findings show the potential of aromatherapy for positive conditioning of odor with a desirable effect. This potential is already known [50, 52] and should definitely be further explored.

#### Consideration of beliefs and expectations

Beliefs and expectations are suspected to play an important role in how users perceive and evaluate aromatherapy [50]. Based on this, a tendency in our results is worth highlighting: The expectations created by (a) the products name and (b) information may have strongly guided the participant’s perception of effects. This is in line with the opinion “that it is the meaning of the aroma that induces the consequent psychological and/or physiological responses” [50] and contains both potential and challenge. Information and product names may open potential by initiating positive expectations, while also minimizing own perceptions by narrowing the room of possibilities. However, whether a positive effect is the result of the essential oils itself or of the underlying beliefs and expectations (or a mixture of both) was of secondary interest in our study context. The possibility of intentionally inducing placebo effects with the help of aromatherapy could be worth a closer look in future research and might have potential on its own.

#### Consider specifics regarding symptomatic burdens and needs

It is well known that health care needs of cancer patients depend on various factors (4). Above all age, type of cancer and treatment, but also side effects of treatment, personal risk factors, individual life situation, and physical and psychological resilience are important (2). All these factors should be considered in aromatherapy interventions. The selection of products should be tailored to the specific symptoms that need to be addressed.

#### Thinking about future challenges

It is important to take typical challenges that symptoms or treatment may cause into consideration. Peripheral neuropathy, for example, well known as a temporary or long-term result of chemotherapy, causes sensory neuropathy, paresthesia, and pain (2). These symptoms can lead to pragmatic problems; opening the products bottles can be difficult or not possible at all, as was the case with some participants in our study. Other challenges, such as breast swelling after mastectomy, experienced by one of our participants after the use of the lavender-based *Relaxing Oil* and *Pillow Spray*, require a more intensive examination. There are already indications in research that lavender influences the hormone balance [53]. It is important to investigate this evidence further, specified to the use of lavender essential oil in women with hormone-dependent cancer.

### Methodological limitations

The mixed-methods design with a predominantly qualitative approach provides only a descriptive overview of the quantitatively collected symptomatic burdens of the participants. For a correlation of the potential of aromatherapy with symptoms, supportive therapies, and side effects, a quantitative design with a larger sample and comprehensive symptom analysis would be required.

A further limitation lies in the evaluation of self-tailored individual applications of heterogenic products: the approach prevents recognizable comparability of essential oils’ effects. As with other complex interventions, the multiple components of individually applied aromatherapy interact with each other and possibly unfold synergistic effects that could not be captured in this analysis [54]. Although the effectiveness of the essential oils cannot be proven within this research design, we were able to show that a great potential of aromatherapy lies **precisely** in the creative and free application that makes the intervention so complex and individual.

Another methodological limitation is to be mentioned concerning our sample: Although we aimed for a sample with maximum variation, all participants with gynecological cancers had ovarian cancer. Thus, we may have missed information about diagnose-related specifics in using and perceiving aromatherapy for other gynecological cancer types. This corresponds to the underrepresentation of women with gynecological cancers in aromatherapy research in general [10] and needs to be addressed in future research.

## Conclusions

As cancer patients and survivors are usually considered very motivated when given the right tools and guidance (1), it is important to offer appropriate tools and support for independent application and use. Ideally, tools should be applicable for everyday life and adaptable to specific needs and circumstances of those affected. With this study, we explored aromatherapy from the user’s point of view. Our findings demonstrated the potential of aromatherapy as a supportive treatment modality that can be used as a kind of toolbox. Aromatherapy shows potential for relieving symptomatic burdens directly — especially nausea, peripheral neuropathy, pain, and sleep. In addition, indirect effects were found to affect symptomatic burdens, by the development and support of coping strategies using aromatherapy. Successful promotion of well-being and mindfulness with aromatherapy was also found. The framework of a study, with the task to use aromatherapy regularly, seemed to give our participants needed motivation and legitimization. At the same time, precisely, the ability to tailor use and individualize applications resulted in a high level of satisfaction. These findings should be applied to future developments of aromatherapy interventions and research designs. Yet challenges, such as individual odor aversions and intolerances, as well as consideration of medication- and disease-related limitations, should be reflected. A more systematic approach toward specific symptoms with focus on quantitative research would be useful to confirm the potential.

## Supplementary information

Below is the link to the electronic supplementary material.

Supplementary file1 (PDF 429 KB)

## Data Availability

Research data are not shared due to privacy or ethical restrictions.

## Footnotes

1 CIM means the combination of evidence-based complementary medicine with conventional medicine (= Integrative Medicine) in a holistic and patient-oriented way (Frass et al., 2019).

2 Detailed presentation of the results in supplement 1 “results”.
