# Supplementary material for: “Like one part of a puzzle” — individualized aromatherapy for women with gynecological cancers in aftercare: results from a qualitative-focused mixed-methods study
Source: Support Care Cancer. 2022 Dec 23;31(1):80. doi: 10.1007/s00520-022-07543-z (PMC9789001; doi:10.1007/s00520-022-07543-z)
Supplement: Supplementary file 1 — Supplementary file1 (PDF 429 KB) [file 520_2022_7543_MOESM1_ESM.pdf]

Results in detail

Twenty participants were initially included in the study. Two dropped out two weeks after the first meeting after the baseline survey with the MYMOP2 questionnaire but before the interview due to (1) private reasons and (2) no reason cited. Eighteen participants completed the study 4 to 6 weeks after the individualized aromatherapy applications with the follow-up survey of MYMOP2 and the qualitative interview.

3.1. Characteristics of the participants and results of the MYMOP2 questionnaire (table 2)

The participants' age distribution ranged between 39 to 77; the majority were between 50 and 60 years old. The participants had breast (n=6), ovarian (n=11), and uterine cancer (n=1). Their last chemotherapy was between 6 months and 7 years ago. For most of the women (n=6), the last chemotherapy was completed in the previous 1-2 years. One woman started with a grade IV chemotherapy after ten days of aromatherapy.

Table 2: Characteristics of the participants

**Participant**	**Age**	**Diagnosis** [ovarian cancer (O), uterus cancer (U), breast cancer (B)]	**Time since diagnosis** [<1, 1-2, 3, 4, 5+ years]	**Therapy**				**Symptomatic Burdens (MYMOP2)**
				Chemotherapy	Surgery	Radiation Therapy	Maintenance Therapy	
**TN_01**	70-80	O	1-2	X	X			1) peripheral neuropathy<br>2) fatigue
**TN_02**	50-60	O	1-2	X	X		PARPi	1) peripheral neuropathy<br>2) intestinal complaints
**TN_04**	50-60	O	1-2	X	X		PARPi	1) fatigue<br>2) peripheral neuropathy
**TN_05**	60-70	U	4	X	X	X		1) pain<br>2) intestinal complaints
**TN_06**	70-80	O	5+	X	X			1) peripheral neuropathy<br>2) intensified odor sensation
**TN_07**	60-70	O	1-2		X	X		1) pain<br>2) peripheral neuropathy
**TN_08**	60-70	B	<1	X	X	X		1) peripheral neuropathy<br>2) fatigue
**TN_09**	40-50	B	4	X	X			1) fatigue<br>2) anxiety
**TN_10**	50-60	O	<1	X	X		VEGFi	1) rheumatism<br>2) concentration problems
**TN_11**	50-60	B	4	X	X	X		1) sleeping disorders<br>2) temper
**TN_12**	60-70	O	3	X	X			1) fatigue<br>2) intestinal complaints
**TN_13**	50-60	O	5+	X	X		PARPi	1) depression<br>2) peripheral neuropathy
**TN_14**	60-70	O	3	X	X			1) sleeping disorders<br>2) fatigue
**TN_15**	40-50	O	3	X	X			1) peripheral neuropathy<br>2) depression
**TN_16**	50-60	O	3	X	X			1) peripheral neuropathy<br>2) depression
**TN_18**	18-40	B	5+	X	X		X	1) fatigue<br>2) dry mucous membranes
**TN _19**	50-60	B	1-2		X		X	1) hot flushes<br>2) sleeping disorders
**TN_20**	50-60	B	1-2		X		X	1) pain<br>2) sleeping disorders

The most relevant, therapy-related symptoms listed by the participants in the MYMOP2 questionnaire were peripheral neuropathy (n=8), fatigue (n=8), sleeping disorders (n=4), and pain. In addition, women listed anxiety, depression, dry mucous membranes, hot flashes, intensified olfactory perception, intestinal complaints, and aggravation of rheumatism (figure 1). Activities that are complicated by the named symptoms are shown in figure 2. Most women indicated a general deterioration of their social life. Specific individual symptoms were documented under the category "other", and are related to nutrition, studying, or activity limitations in general.

The follow-up MYMOP2 data were based on the perceptions of the participants about the same two symptoms and one limited activity they have already indicated and assessed in the baseline survey. During the 4–6-week application period, the everyday life of many participants may have changed due to vacation, reduction of working hours, new diagnoses, or new medication among other factors. In addition to aromatherapy, these changes may have also affected the perceived burden of the symptoms. Hence, the MYMOP2 results should only be considered as an initial overview over the effects of aromatherapy on the participants' symptoms. Considering this background, the overall changes in symptom burdens (figure 3) and in limitations of activities (figure 4) show a tendency towards improvement.

This initial outlook is augmented and contextualized by the qualitative data from the semi-structured interviews, which each lasted between 18 and 80 minutes.

Figure 1: Symptom burden (MYMOP)

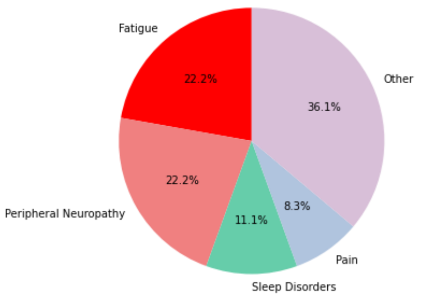

Figure 2: Limited activities

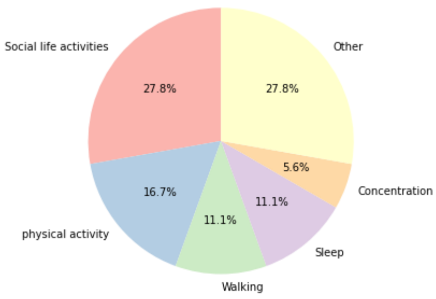

Figure 3: Change of symptomatic burdens

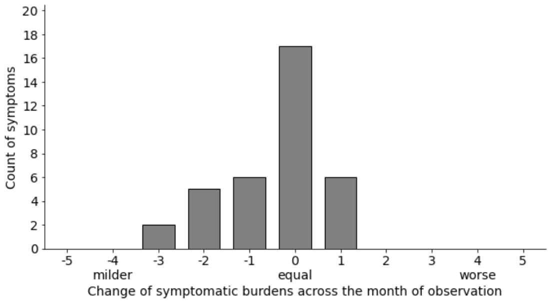

Figure 4: Change in activity limitations

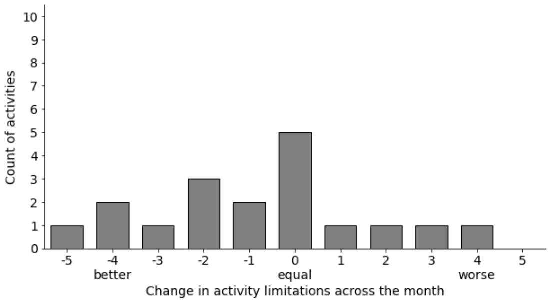

3.2. "It's one part of a puzzle" – Individual experiences, perceptions on individualized aromatherapy

Compared to the MYMOP2 survey results, the qualitative data show a broader and deeper picture of essential-oil use. Not only the potential for treatment beyond improving symptom burden, general wellbeing, and activity limitations, but also the different ways that aromatherapy can be used, and the challenges, the experiences, and the accompanying attitudes.

3.2.1. Using aromatherapy: from patterns to individual tailoring

Expectations and experiences in getting started with aromatherapy

The participants had different needs, requirements, and expectations for aromatherapy. Most had had previous experiences with essential oils used as wellness products. High expectations for symptom relief through aromatherapy were hardly mentioned explicitly. However, in some cases, implicitly expressed disappointment signaled a high level of expectation. Nevertheless, almost all participants were positively oriented toward aromatherapy. A few of them took a more neutral and even skeptical stance. Accordingly, different approaches can be identified in the way the essential-oil products are tested and gradually embedded in everyday life: While some of the participants developed clear structures and routines, most of the women preferred to use the products flexibly. It appeared to be vital for most of the participants, however, to use the essential-oil products in their own way, adapted to their needs and subjective preferences. The brochure with information on the products and application examples was found to be a good introduction by all participants. Nevertheless, three types of reactions could be differentiated: 1) welcoming the open concept to test and explore, 2) desiring more concrete instructions, such as precise details on the type, time, frequency and aim of the applications, and 3) using the information to gain a first overview over the topic that motivated them to further research about aromatherapy independently.

Characteristics in the use of aromatherapy

The reasons for use were extremely diverse ranging from specific and non-specific (table 3).

Table 3: Reasons to use aromatherapy

Main-codes	Sub-codes	Exemplary quotes
**Non-specific reasons**	General improvement of well-being<br>→ Doing something good (1)<br>→ "Just because I like the smell" (2)<br>→ Undirected reasons (3)	(1) *So, when you're in the situation with cancer, then […] you naturally pull at all straws and I just try to do something good for myself. And I have to say that for me this was very, very pleasant. (TN01)*<br>(2) *[I] use it […] because I just love the smell. (TN02)*<br>(3) *[I] thought, just take it out right now, just do it and also do it differently, I would say. I can't now say how, when, but I used it very often. (TN01)*
**Specific reasons "Means to an end"**	**Relief of symptom burden**<br>→ Symptoms reported in MYMOP (sleep (1), fatigue, peripheral neuropathy, anxiety/depression)<br>→ Other symptoms (nausea, pain (2), skin- and scar-car)<br>**Coping strategies for every-day challenges**<br>→ Activation (e.g., substitute for coffee (3), rise energy level)<br>→ Stabilization<br>→ Relaxation<br>→ Distraction<br>→ Refreshment<br>→ Concentration<br>→ Mark and support transitions<br>→ Support (4)	(1) *The pillow spray. I actually used that often, because I noticed that it helped me to fall asleep. (TN20)*<br>(2) *And I used it [Peppermint, JC] partially, I think, for massaging the places that had pain; I massaged it in. (TN20)*<br>(3) *The Energy Kick has slowly replaced coffee, at least it has the character of a ritual, I think. (TN08)*<br>(4) *With migraines it gives a little support in the way that I could say that the migraine isn't so sharply painful. I don't know if it's possible to understand what I mean. (TN13)*

Place, frequency, and type of use[1]

Participants used aromatherapy mostly at home and at work, but also (some) mentioned using it while travelling and on holiday. The frequency of use varied greatly between the participants; however, the exceedingly frequent establishment of routines and regular use is worth mentioning. In some cases, these routines led to positive conditioning, whereby, the mere thought of aromatherapy led to an improvement in well-being and the

[1] Summarized in table 4, main text.

feeling of being supported: *"Ah yes, feel supported. I feel fortified with the thought alone that I'm taking part in this study. Yes, […] the thought alone was helpful"(TN09).* Applications with essential oils were used percutaneously or olfactorily. While applications via the skin were often combined with massages, the olfactory applications were used by scenting in the air, or through scenting via a carrier object. The choice of scent-carrier object varied: pillows, curtains, handkerchiefs, masks, odor-sticks, and candles were used. In any case, creative or gamified ways of using aromatherapy scents were also highlighted by participants. For instance, one participant developed a method to promote mindfulness by exposing herself to random scent stimuli with *Peppermint* and *Good Mood* in unlabeled scent sticks. Also, the social environment was sometimes included in creative games with essential oils: for example, in scent guessing games and tests, where the scent was not initially named in order to sharpen olfactory perception or to match others' reactions with their own.

Motivation to use aromatherapy[2]

Underlying motivations may be of great relevance for the consistent use of the essential oils over the entire intervention period – and beyond. Although the participants reported different reasons for taking part in the project, two key motivations could be identified: First, the study participation entailed a <u>commitment</u> on the part of the participants. Getting involved with the essential oils and practice aromatherapy in a regular way was described by some women as an indulgence and luxury that they would not have allowed themselves outside the task given within the study participation. Described as *"this artificial binding of the task" (TN16),* the assignment gave them the legitimacy to do something for themselves. It <u>legitimized</u> them to act in their own self-interest, and to take time for self-care: *"But the stimulus of this study […] clearly released the feeling in me, that I have all the time in the world to intensively pursue and use these alternatives" (TN12).* Second, the framework of the study gave many of the participants the needed impetus for trying out new daily and personal uses of aromatherapy. Words such as euphoric, thrilled, enthusiastic, curious were used by participants to describe their motivation for experimenting. Furthermore, some participants supported their initial motivation actively with established routines.

3.2.2. Participants view on the potential of individualized aromatherapy

From our analysis we found potential, challenges, and a co-existence of both in dealing with aromatherapy.[3]

The identified key potential of individualized aromatherapy can be summed up in its successful use as a tool for promoting self-care in different areas and with different approaches: for symptom control, promoting well-being, encouraging mindfulness, and developing strategies:

Aromatherapy as a tool for symptom control

Participants reported aromatherapy-associated improvements in symptomatic burdens they specified in the MYMOP2 questionnaire, such as sleeping disorders, fatigue, nausea, pain, and various manifestations of peripheral neuropathy. In the interviews the improvement was mostly described as moderate improvement and often closely linked with increased mindfulness and a general intensified focus on self-care. Particularly, peripheral neuropathy was treated effectively with massage (Relaxation Oil) by some participants and they reported (a) strong relief from cramp-like complaints and (b) tingling sensations in numb limbs. Other direct symptom relief was also mentioned: One participant stated that she relieved her drug-induced persistent nausea with peppermint oil for a few hours at a time after application: *"The peppermint oil I use three times a day, always when I notice that I feel nauseas and then I feel free of nausea. So even now it still always works." (TN02).* In addition, women referred to improvements of specific symptoms not mentioned in MYMOP2, such as headaches, muscle pain, skin- and scar-care. They used the essential oils successfully to relieve these discomforts, often in combination with conventional treatment like pain-medication or other strategies, such as cold compresses, resting in the dark, or massaging the temples, as clearly shown in this quote: *"It really calmed me a bit, when I could say: Okay, relasx, relax. So, I would need a little less of the medication […]. So, I found that very pleasant." (TN13).* This participant described using aromatherapy for reducing her pain medication intake through the calming-down effect she has experienced. This view opens the door to the more holistic means of aromatherapy that influence well-being and mindfulness:

[2] Summarized in table 4, main text.

[3] Summarized in table 5, main text.

Aromatherapy as a tool to promote well-being

"And even now I am convinced that it is one piece of the puzzle that can enable relaxation, letting go, calm, or anything else in this direction" (TN12).

Beyond symptom control, the participants reported the potential of essential-oil use to promote their well-being. In this regard, they referred to the effects of aromatherapy on energy and emotions that they perceived. For instance, by developing strategies for the essential-oils to influence their energy-level to 1) relax and calm down, and 2) activate, invigorate, and refresh. For the first purpose, the lavender-based products (*Pillow Spray, Relaxing Oil*) were mostly used – often in conjunction with ritualized procedures and massages. In contrast, to increase energy, the products *Energy Kick, Good Mood*, and *Peppermint* were mostly used.

Also, but rarely, other products were used to calm down and relax, when participants recognized that a specific odor, triggered calming memories in them: *"Peppermint is a smell that for me is very positively-associated with my grandmother. And even now I use it […] often and constantly, because it calms me. As I said, because there's so much past there" (TN13).* The work with the essential-oil products helped participants to influence and improve their mood and emotions. Participants reported a feeling of security, increased optimism and good mood, feelings of happiness, and general mood-enhancing effects through aromatherapy. However, participants were not always able to specify their perceptions concerning their essential-oil use. In this case, they described their experiences in rather general terms. In doing so, they spoke of an improvement of their general well-being, of pleasant and beneficial effects, without further differentiating. *"And you feel that you [do] something good for yourself. Yes" (TN09),* is a quote that aptly expresses the unspecified positive statements that pervade many descriptions of perceived effects of aromatherapy.

Aromatherapy as a tool to encourage mindfulness

Participants used aromatherapy to successfully set an olfactory marker and consciously enter the present moment. For this, they anchored in the scent of the essential oils. *"It's like an anchor" (TN 09),* one participate specified. This anchoring function was used by the participants for various reasons. For instance, to change unwanted situations (such as by transforming feelings of anger or stress to relaxation) or strengthen desired emotional states (such as concentration or relaxation) by setting a focus with the odor. Specifically, participants described how they used essential oils to give a protective (odor) cover to feel more secure (for instance, when the illness made them feel weak), to create personal space, consciously shift the focus through odor triggers, or to motivate. The metaphor, *"it's like an anchor"* refers to the supporting potential of aromatherapy – particularly the olfactory elements of it – to keep the mind anchored in the scent, hence, to create and establish mindfulness in everyday-life situations

Aromatherapy as a tool to develop strategies

In addition, another type of potential could be identified: aromatherapy was used by the participants to develop strategies that support them in their daily life. In a sense, aromatherapy has been used as a tool to develop new strategies or to support existing ones: Some participants established new strategies – for example routines based on essential oil applications – others used aromatherapy in conjunction with already existing coping-strategies such as meditation, medication, mindfulness-training, to strengthen positive developments in their care or as an assistive device to soften (illness related) challenges in everyday life and to be able to do something about it on their own: *"There the [radiation, JC] is working internally, and then to do something about it that can work against that, like these beautiful scents, it does have a [soothing] effect." (TN08)*

Ambiguity of prior information: potential and challenge at the same time

Overall, there is a tendency that the expectations created by (a) the product name (*Energy Kick, Relaxation Oil, Good Mood*) and (b) the information given in advance (for example from the brochure) may guide the perceived effects in specific directions. That can be seen (and used) as an opportunity, in the sense of reinforcement of desired effects, as the following quote exemplifies: *„So, I also liked this fragrance, I liked the smell of it, and I found it pleasant and then it said on it ‚Energy' and I thought - I need energy now, so I will take energy. And then I felt energy" (TN04).* On the other hand, it might simultaneously be a challenge, because the information and product names could hinder unexpected positive effects from being recognized. What is about other, more obvious, challenges and impediments of aromatherapy use?

3.3.3. Challenges of aromatherapy

Although all participating women reported an overall positive impact of the individualized aromatherapy, some challenges were reported. The most serious effect reported was the association of the lavender-based products (*Pillow Spray* and *Relaxing Oil*) with swelling of both breasts after mastectomy in one participant. As a result,

the lavender-based products were reduced by the participant until the swelling decreased. Another patient with therapy-related sensitive mucosa nose-skin stopped using the essential-oil products after three weeks – except *Relaxing Oil* – due to irritated nasal mucosa.

Other reported challenges were related to: (a) difficulties in opening the bottles by some women with sensory restrictions in the hands due to peripheral neuropathy; (b) lack of time to use the products regularly; (c) aversion of the odor/s by family members; (d) olfactory aversions by the participants themselves. Aside from this, challenges due to (unconscious) expectations of what effect aromatherapy would have, appeared. These were expressed by the disappointment when the participants experienced either no effects at all or even unwanted effects. The latter refers to participant-reported negative associations after exposure to the fragrance, and aversion to the scent that triggered nausea in two cases or feelings of stress in another case. Both were connected to the *Pillow Spray*.

3.3.4. Interim conclusion

Despite the challenges presented above, and although not all participants were able to report improvement in their specific symptoms, they all had a positive overall summary of their experiences with the essential oils – each with its own unique explanation. On this basis, it may be concluded that aromatherapy within the targeted group have more holistic potential than mere symptom control. One participant perceives it as a one piece of a greater puzzle that can change the whole situation for the better: *"And I am still convinced, that this is one part of a puzzle …" (TN12).*
